# Research needs on the biodiversity–ecosystem functioning relationship in drylands

**DOI:** 10.1038/s44185-024-00046-6

**Published:** 2024-06-05

**Authors:** Fernando T. Maestre, Lucio Biancari, Ning Chen, Mario Corrochano-Monsalve, G. Darrel Jenerette, Corey Nelson, Kaarina N. Shilula, Yelyzaveta Shpilkina

**Affiliations:** 1 Environmental Sciences and Engineering, Biological and Environmental Science and Engineering Division, King Abdullah University of Science and Technology, Thuwal, 23955-6900 Kingdom of Saudi Arabia; 2 IFEVA, Universidad de Buenos Aires, CONICET, Facultad de Agronomía, Av. San Martín 4453, Buenos Aires, C1417DSE Argentina; 3 Cátedra de Ecología, Departamento de Recursos Naturales y Ambiente, Facultad de Agronomía, Universidad de Buenos Aires, Av. San Martín 4453, Buenos Aires, C1417DSE Argentina; 4 Instituto Multidisciplinar Para el Estudio del Medio “Ramon Margalef”, Universidad de Alicante, Carretera de San Vicente del Raspeig s/n, 03690 San Vicente del Raspeig, Spain; 5 State Key Laboratory of Herbage Improvement and Grassland Agro-ecosystems, College of Ecology, Lanzhou University, No.222, Tianshui South Road, Lanzhou, Gansu 730000 China; 6 Departamento de Genética, Antropología Física y Fisiología Animal, Facultad de Ciencia y Tecnología, Universidad del País Vasco (UPV/EHU), Leioa, Spain; 7 Department of Botany and Plant Sciences, University of California, Riverside, CA USA; 8 Departamento de Ecología, Universidad de Alicante, Carretera de San Vicente del Raspeig s/n, 03690 San Vicente del Raspeig, Spain

**Keywords:** Biodiversity, Community ecology, Ecosystem ecology

## Abstract

Research carried out in drylands over the last decade has provided major insights on the biodiversity–ecosystem functioning relationship (BEFr) and about how biodiversity interacts with other important factors, such as climate and soil properties, to determine ecosystem functioning and services. Despite this, there are important gaps in our understanding of the BEFr in drylands that should be addressed by future research. In this perspective we highlight some of these gaps, which include: 1) the need to study the BEFr in bare soils devoid of perennial vascular vegetation and biocrusts, a major feature of dryland ecosystems, 2) evaluating how intra-specific trait variability, a key but understudied facet of functional diversity, modulate the BEFr, 3) addressing the influence of biotic interactions on the BEFr, including plant–animal interactions and those between microorganisms associated to biocrusts, 4) studying how differences in species–area relationships and beta diversity are associated with ecosystem functioning, and 5) considering the role of temporal variability and human activities, both present and past, particularly those linked to land use (e.g., grazing) and urbanization. Tackling these gaps will not only advance our comprehension of the BEFr but will also bolster the effectiveness of management and ecological restoration strategies, crucial for safeguarding dryland ecosystems and the livelihoods of their inhabitants.

## Introduction

**Fig. 1:**
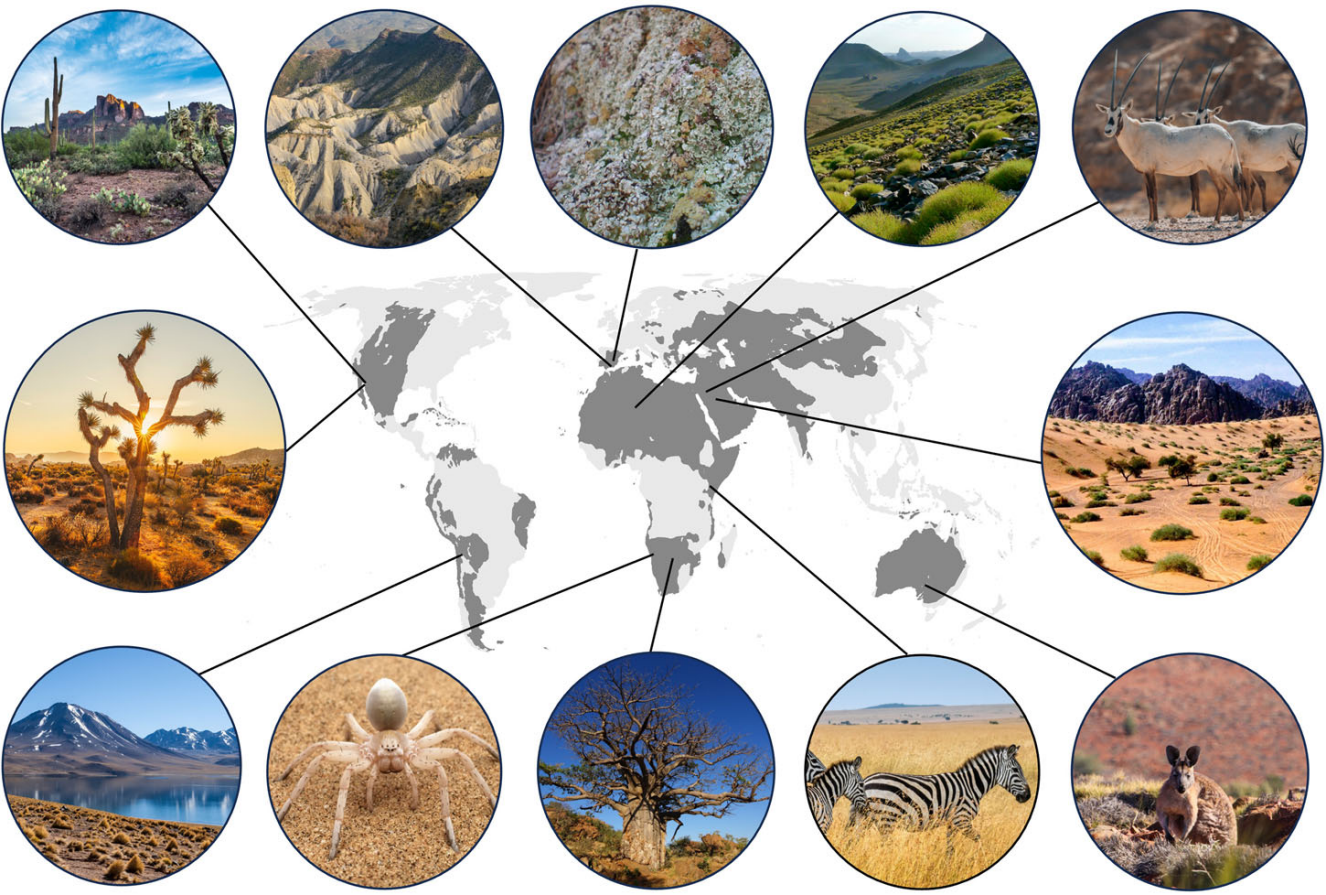
Examples of representative dryland biota and landscapes. Drylands areas are marked in dark gray in the map. Photo credits (from left to right and from the upper to the lower part of the figure): Raychel Sanner on Unsplash, Leo Barco, Fernando T. Maestre, Azzedine Rouichi on Unsplash, NEOM on Unsplash, Explore with Joshua on Unsplash, Rabah Al Shammary on Unsplash, David Vives on Unsplash, Markus Blüthner on Unsplash, Leon Pauleikhoff on Unsplash, sutirta budiman on Unsplash, and Megan Clark on Unsplash.

Drylands are broadly defined as those areas with an aridity index (precipitation/potential evapotranspiration) below 0.65^1^. They host a biota that must cope with environments characterized by the scarcity of resources such as water and soil nutrients, highly variable and in some cases extreme climatic conditions, and recurrent disturbances such as droughts^2^. Contrary to the belief that these harsh environmental conditions should restrict biodiversity (e.g., the environmental filtering theory^3^), drylands host unique landscapes and biota (Fig. 1) that have fascinated explorers and scientists for centuries^4–6^. As an example, drylands show a high diversity of plant leaf functional traits mirroring that observed across the rest of terrestrial ecosystems (the so–called drylands functional paradox^7^). Other remarkable findings are that dryland vegetation has been found to contribute disproportionately to observed global productivity increases over the last decades^8^, and that iconic dryland megafauna, such as elephants, not only are fundamental for the development of local communities but also contribute to soil carbon sequestration^9^. These discoveries have helped to increase awareness among scientists and the general public of the importance of drylands and their biodiversity for sustaining life on our planet as we know it, and for mitigating the impacts of ongoing climate change.

A key topic of biodiversity research over the last 30 years has been to understand the functional consequences of biodiversity, and in particular the relationship between biodiversity and ecosystem functioning (BEFr hereafter)^10,11^. This body of work has, among other aspects, shown that increases in both species richness and functional diversity enhance key ecosystem functions such as productivity across a wide range of ecosystems, has revealed the mechanisms behind the BEFr, and has shown that diversity effects increase through time^10^. While most of the initial research on this topic was carried out in non–dryland environments, over the last decade dryland research has made fundamental contributions to our understanding of the BEFr. These include, among others, the first empirical evidence at the global scale of positive links between plant^12^ and microbial^13^ diversity and ecosystem multifunctionality (EMF), assessing how multiple biodiversity facets (taxonomic, functional, and phylogenetic) jointly affect EMF globally^14^, and highlighting how functional diversity maximizes EMF across drylands worldwide^15^.

In recent years, BEF research is moving from demonstrating that biodiversity matters for ecosystem functioning to understanding the context–dependency of the BEFr and to explicitly account for the inherent complexity of dealing with multi–taxa and multi–trophic ecosystems that change both in space and time. This includes evaluating how the BEFr is modulated by other biotic and abiotic factors^16^, how it changes through time^17^ and depends on the functions being considered^18^, or how diversity across multiple taxa and trophic levels impact the BEFr^19^. Despite increased research efforts over the years, some of these emerging topics have not been addressed in drylands yet or are just starting to be explored^20,21^.

**Fig. 2:**
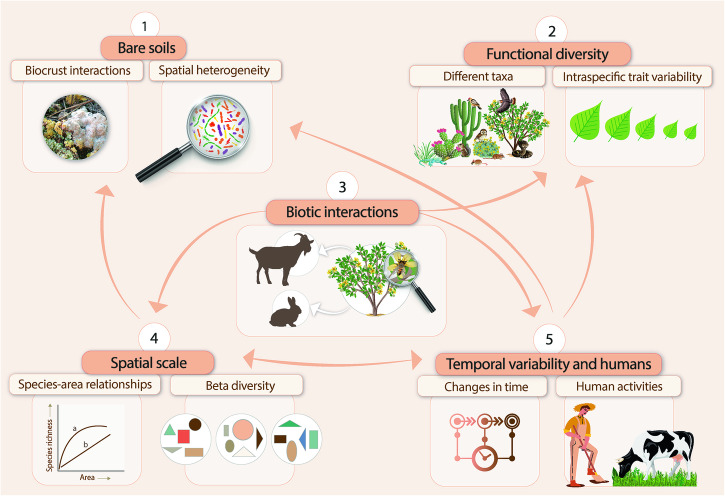
Knowledge gaps about the biodiversity–ecosystem functioning relationship (BEFr) in drylands discussed in this article. These include addressing the BEFr in bare soils and evaluating the influences of various factors such as biotic interactions, trait variations, species–area relationships, beta diversity, and human activities on the BEFr. The numbers correspond with the order in which these gaps are presented in the abstract and the main text. The arrows show connections between the different gaps, and aim to highlight how advances in our understanding of a given topic can contribute to fill other gaps in our understanding of the BEFr.

Here we discuss some emerging and understudied questions about the BEFr in drylands (Fig. 2). We do not pretend to provide an in–depth review of all the relevant topics surrounding the BEFr, but rather to highlight a few key knowledge gaps that, in our opinion, need to be addressed by future research to better understand this relationship in drylands. In doing so, we can not only deepen our understanding of the BEFr, but also propel research efforts aimed at conserving biodiversity and effectively managing ecosystem services vital for supporting the livelihoods of over 2 billion people residing in dryland regions, which cover more than 41% of the Earth’s surface^22^.

## Bare soils, a key but understudied feature of dryland ecosystems

**Fig. 3:**
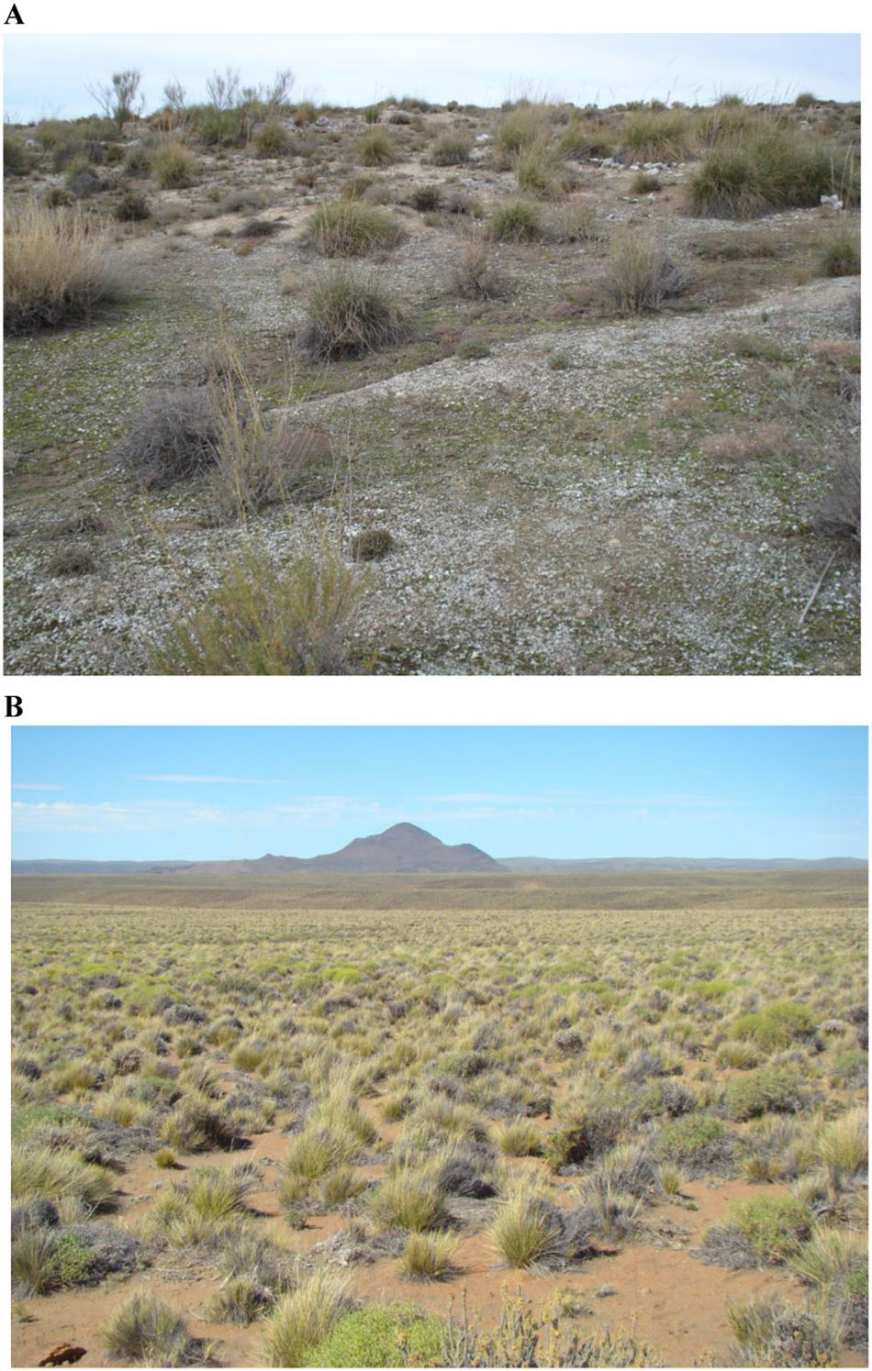
Examples of the two-phase mosaic of discrete plant patches surrounded by a matrix of bare soil typically found in drylands worldwide. The figure shows examples of bare soils with (**A**) and without (**B**) well-developed biocrusts. See Supplementary Fig. 1 for close-up examples of the types of biocrusts that can be found in drylands. Photo credits: Andrea Castillo-Monroy (**A**) and Juan Gaitán (**B**).

Bare soils, which cover approximately 35 million km^2^ worldwide^23^, are a common and distinctive feature of drylands (Box 1). Dryland ecosystems are typically characterized by discrete plant patches surrounded by a matrix of bare ground devoid of perennial vegetation (Fig. 3), which may or not contain biocrusts (communities of mosses, lichens, cyanobacteria, and other microorganisms living in the soil surface that are prevalent in global drylands; see ref. ^24^ for a formal definition and Supplementary Fig. 1 for examples). Despite their global extent, bare soils have been largely overlooked in the BEFr literature compared to vegetated areas^10,11,25^.

Bare soils are highly exposed to abiotic factors because they lack the buffering effect of vegetation or biocrusts^26^. In this sense, these areas represent an excellent opportunity to explore BEFr under extreme edaphoclimatic conditions, which are forecasted to extend in future decades^27^, and can shed light on mechanisms that can be relevant for understanding ecosystem responses to climate change in non–dryland environments^28^.

Our understanding of the BEFr in bare soils is significantly limited when compared to our extensive knowledge of soils with vegetation, and to a lesser degree, biocrusts^29^. We could hypothesize that a very plastic BEFr can occur in these areas, with a microbiome adapted to an efficient –but also fast– use of scarce resources resulting in great metabolic pulses linked to rainfall events^30^. A very specialized microbiota might be necessary for doing so. However, we lack sufficient data to generalize whether a higher or a lower diversity is needed in bare soils to maintain ecosystem functioning. The bare soil microbiome might contain a rich batch of genes associated with efficient resource use and resistance genotypes, of which a majority are probably not available in public datasets^31^. In the absence of vegetation, microbially mediated modifications of soil properties and their influence on nutrient cycling could become even more relevant^32^. We have very limited field data regarding nutrient transformation rates in these areas. Some studies suggest intense leaks of nitrogen in gaseous forms in arid environments^33,34^, but the role of biodiversity in influencing such emissions remains almost unknown and represents a key topic for future research.

Understanding the BEFr of bare soils can also be helpful to enhance the success of ecological restoration initiatives. Gaining insights into the intricacies of the BEFr in bare soils would provide valuable knowledge to determine whether a given bare soil has the microbial species and interactions needed to maintain nutrient cycling^35^, which is essential for the survival of tree/shrub/grass plantations and thus to support essential functions and services.

Box 1 What are bare soils in drylands? Expanses of open ground dotted with sparse vegetation are a common feature of dryland ecosystems (Fig. 1). This spatial heterogeneity typically gives rise to soil microsites with varying biodiversity and functioning^2,29,70^. Here, we broadly define three microsites that are commonly found in drylands worldwide as: i) soils influenced by the canopy and nutrient inputs of perennial vegetation, ii) bare soils inhabited by biocrusts (typically found in plant interspaces, see Figs. 3 and S1), and iii) bare soils, which are those not directly influenced by aboveground parts of perennial vegetation nor harboring developed biocrusts. Investigations of soil biodiversity and their relationship to ecosystem functioning in drylands tend to focus on soils influenced by perennial phototrophs (vegetation and biocrusts), while bare soils have typically been understudied.

## Accounting for understudied facets of functional diversity

Functional traits represent the physical, chemical, physiological, structural, phenological, or behavioral attributes of organisms that have an impact on their performance, overall fitness and contribution to ecosystem processes^36,37^. They are commonly used to characterize community responses to environmental changes and to measure how shifts in communities affect ecosystem functions^36,37^. As such, the study of functional traits can help us better understand the mechanisms underlying the BEFr^36^ and holds particular significance in drylands owing to the distinct features of these ecosystems. These include a greater plant functional diversity and a higher evenness in the distribution of plant functional traits compared to other biomes^7^. Indeed, the importance of functional diversity for the BEFr in drylands has been already demonstrated using multiple spatial scales and taxa^38,39^.

Intra–specific trait variability has been shown to play a substantial role in shaping the response of certain plant functional traits to aridity in drylands^38,40^, and its role in maintaining ecosystem functioning is being increasingly recognized^41^. However, there is a lack of studies addressing the impact of such variability in the BEFr in drylands and thus it is largely unknown how the loss of intra–specific trait variability could affect the functioning of these ecosystems. Such loss can have important functional consequences, as genetic erosion and the loss of phenotypic diversity could result in a decrease of ecosystem functioning without losing species from an ecosystem^42^.

Research on the ecological roles of functional diversity in general, and on the BEFr in particular, has largely focused on the study of plants (see ref. ^43^ and references therein). Being crucial for ecosystem functioning, plants mainly operate within a single trophic level, and thus an excessive dependence on plant traits tends to overlook the intricacies and significance of functional diversity across multiple trophic levels^44^. The analysis of functional traits of biocrusts is receiving increasing attention over the years^39,45,46^, whereas comparatively less emphasis has been placed on exploring the functional traits of animals^44^. Furthermore, no previous study has, to our knowledge, assessed how the functional diversity of biocrusts and animals impact the BEFr in drylands, and thus the role of the functional diversity (vs. taxonomic diversity) of these important organisms for the maintenance of ecosystem functioning in drylands is unknown. This is thus an important knowledge gap to be covered by future studies.

## Biotic interactions, critical drivers of dryland ecosystem structure

Species in drylands are adapted to exist at the edge of environmental conditions suitable for life^2^. The distribution of these organisms is subject to biotic interactions, which largely influence the structure and functioning of these ecosystems^7,47^. For example, many dryland species depend on positive biotic interactions for their persistence in stressful water–limited environments^48,49^. Further, observed positive effects of biodiversity on ecosystem functioning lie on the basis of a positive balance between negative (e.g., competition) and positive (e.g., facilitation and mutualism) interactions^50^. Understanding the influence of biotic interactions on the BEFr is crucial as species interactions may be more prone to rapid shifts due to the increased frequency and magnitude of extreme events in drylands^51^.

Some key questions remain to be elucidated regarding plant–animal interactions and the BEFr that are particularly important in drylands. We know very little about how interactions between plants and soil fauna affect ecosystem functioning and the BEFr in these areas, but they will likely play an important role. For example, in Neotropical savannas, the composition and diversity of soil epigeic fauna have been found to influence litter decomposition rates^52^. It is also known that the release of organic compounds by roots, among other functions, serve to attract beneficial soil invertebrates and disrupt harmful bacteria communication, ultimately fostering plant growth across diverse ecosystems^53^. This area strongly warrants future research attention, together with the study of the relationships between pollinator diversity and ecosystem functioning in drylands, which remain inadequately understood^54^.

Within drylands, biocrusts are biodiversity/functional hotspots that provide a variety of services such as nutrient inputs, stabilization, and alteration of hydrological properties of the soils they inhabit^24,55^. Despite their recognized importance and the growing interest in biocrust BEFr research^29^, investigations into the role of biotic interactions within biocrusts or how these interactions might influence the BEFr in drylands are limited^47^. Recent advances in the microbial ecology of biocrusts have provided evidence that microbe–microbe interactions can influence the BEFr in drylands. For example, the formation of biocrusts typically depends on mutualistic resource trading relationships between pioneer cyanobacteria and soil diazotrophic bacteria during the initial colonization of bare soils to provide nutrient inputs required for the biomass necessary to stabilize soils for further colonization^56,57^. Conversely, a newly described predatory bacterium (*Candidatus* ‘Cyanoraptor togatus’) preys preferentially on specific biocrust–associated cyanobacteria^58^, disrupting the spatial organization of biocrust and creating niches for less abundant –but predation–resistant– biocrust pioneer species, such as those found in the family Coleofasciculaceae (formerly *Microcoleus steenstrupii*)^59^. These biotic interactions fundamentally impact biodiversity and functional traits in biocrust communities at the cm scale, which, given the extent of biocrusts across global drylands^24^, are likely to contribute significantly to ecosystem functioning.

A comprehensive exploration of microbial interactions governing soil processes and how they contribute to the BEFr in drylands can certainly enhance our comprehension of biocrust responses to global change drivers. Predicted climate change will likely have a large effect on biocrusts and the interactions within their constituents, as it has already been shown that temperature and precipitation patterns have differential effects on biogeographical predominance of pioneer soil microbes linked to biocrust formation^60^. Determining whether fundamental microbial interactions can persist under increased frequency and magnitude of extreme events will be key in determining the distribution and functioning of biocrusts in a rapidly changing world. Doing so could not only potentially indicate environmental thresholds that could predict sudden changes in ecosystem functioning as biotic interactions shift, but also aid with the development of soil conservation and restoration strategies in a more arid world.

## Incorporating spatial scale

While much success in BEFr research has been made in plot–scale studies, a need remains to move beyond individual plots to identify landscape interactions with the BEFr^61,62^. This is especially true in drylands because spatial heterogeneity is extensive in these environments^2,7^ and the BEFr may be affected by the distribution of habitat patches and environmental gradients throughout a landscape^63^.

While variation across scales has been considered in detail for biodiversity, we need better approaches for connecting this research to ecosystem functioning. For example, species–area relationships have been considered one of the few “laws” of ecology^64^ but this relationship has had only limited connections to ecosystem functioning^62^. How differences in species–area relationships are associated with ecosystem functioning remain a key question for future BEFr research in drylands. In another example, extensive research has been directed to characterizing beta diversity, or the turnover of species among locations^65^. In contrast to the extensive research on BEFr with plot–scale diversity, more research is needed to characterize how variation in beta diversity affects ecosystem functioning^66,67^.

Through the consideration of scale, the spatial heterogeneity characterizing dryland landscapes should be a key component of landscape controls on the BEFr. Dryland BEFr should vary throughout a landscape in response to variation in water availability, nutrients, and community assemblages, and is likely to be affected by the connectivity among locations and the isolation of habitats^63,68^. How the movement of materials and organisms among habitats (e.g., isolated plant patches) can affect the BEFr is an important frontier for future research^69^. Finally, landscape heterogeneity may reflect self–organization of ecological interactions, which can affect both biodiversity and ecosystem functioning. Two prominent dryland examples, the generation of islands of fertility^70^ and of fairy circles^71^, both reflect an organization of species and ecosystem dynamics that may affect the BEFr.

## Accounting for temporal variability and humans

The structure and functioning of drylands is largely impacted by temporal changes in key resources such as water^2^. Thus it is not surprising to see the large body of conceptual and empirical research aiming to understand the ecological roles of rainfall pulses, which are inherently temporally variable in drylands^30,72,73^. Some studies have addressed how biodiversity, alone and in interaction with other abiotic and biotic factors, impacts ecosystem stability in drylands^20,74^. However, there are still large uncertainties regarding the role that the BEFr plays in the temporal dynamics of these ecosystems, which should be addressed by future research. Key questions to be addressed include: i) evaluating temporal changes in the BEFr at different scales, from rainfall pulses to decadal drought as well as directional changes associated with ongoing warming, ii) explicitly considering both contemporaneous and legacy effects^75^ of temporal variability on the BEFr, and iii) addressing the role of rare places and rare events (“hotspots and hot moments” *sensu* ref. ^76^) on the BEFr.

Another key control on the BEFr in drylands that we should also explicitly consider is the role of human activities, both present and past, which can vary across spatial and temporal scales and have pronounced effects on the structure and functioning of drylands^77–79^. Spanning agricultural practices, from irrigated crops to grazing, development, from urbanization to nutrient pollution, and natural resource management, from fire prevention to restoration, human activities generate novel ecosystems that are likely to have distinct BEFr from theoretically “natural” ecosystems. For example, changes in grazing pressure could modify the effect that plant and herbivore diversity have on the provision of multiple ecosystem services^21^. However, more studies are needed to elucidate the effect of herbivore species replacement (e.g., increasing domestic livestock instead of wild herbivores or vice versa) and on how the diversity (both taxonomic and functional) and complementarity of herbivores affect ecosystem functioning^21^. Studying BEFr in dryland urban environments is also a topic of particular interest. There is a renewed interest and efforts for greening dryland cities to mitigate the impacts of climate change and to increase their livability^80^. These efforts would undoubtedly benefit from studies aiming to understand how the taxonomical and functional diversity of trees and shrubs in urban parks and streets impact water use, evapotranspiration, carbon sequestration, the biodiversity of other taxa, and human wellbeing. Such research holds significant potential to optimize the advantages of greening initiatives while concurrently mitigating water consumption, which is increasingly constrained in drylands globally due to climate change and mismanagement^81^. The imprint of historic land use by humans also has a lasting effect on soil conditions; for example, agricultural activities more than 1000 years in the past have influenced current patterns of desert species assemblages and soil conditions^82^. A better understanding of legacy effects from human activities could also inform future forecasts for drylands, as recently illustrated with the use of climatic legacies for predicting the future distribution of dryland forests^83^.

### Supplementary information

Supplementary Material
