# Supplementary material for: Research needs on the biodiversity–ecosystem functioning relationship in drylands
Source: NPJ Biodivers. 2024 Jun 5;3:12. doi: 10.1038/s44185-024-00046-6 (PMC11332164; doi:10.1038/s44185-024-00046-6)
Supplement: Supplementary file 1 — Supplementary Material [file 44185_2024_46_MOESM1_ESM.pdf]

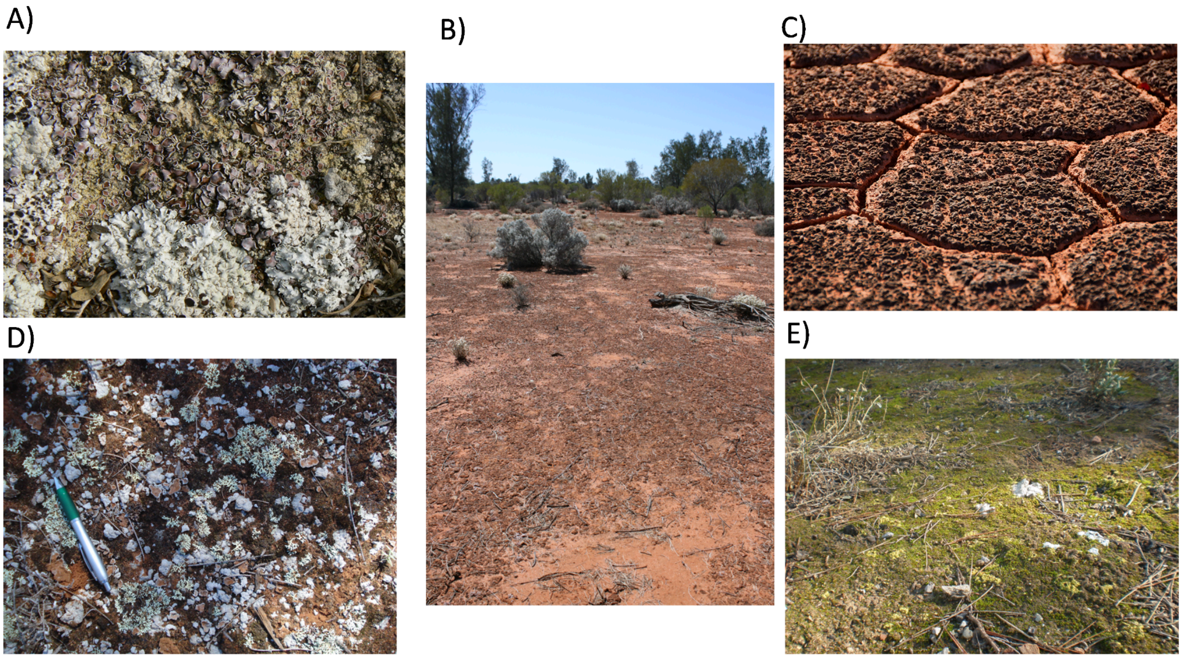

Supplementary Figure 1. Examples of bare ground areas with lichen-dominated cyanobacterial biocrusts (A), cyanobacteria-dominated biocrusts (B, C), cyanobacteria/lichen/moss-dominated biocrusts (D) and moss-dominated biocrusts (E). Photographs by Matthew Bowker (A, C and D), David Eldridge (B) and Fernando T. Maestre (E). Figure obtained from Maestre, F. T. *et al.* The BIODESERT survey: assessing the impacts of grazing on the structure and functioning of global drylands. *Web Ecology* **22**, 75–96 (2022).
